# Inferring differential protein binding from time-series chromatin accessibility data

**DOI:** 10.1093/bioadv/vbaf080

**Published:** 2025-04-10

**Authors:** Sneha Mitra, Alexander J Hartemink

**Affiliations:** Department of Computer Science, Duke University, Durham, NC 27708-0129, United States; Department of Computer Science, Duke University, Durham, NC 27708-0129, United States; Program in Computational Biology and Bioinformatics, Duke University, Durham, NC 27710, United States

## Abstract

**Motivation:**

Due to internal and external factors, the epigenomic landscape is constantly changing in ways that are linked to changes in gene expression. Chromatin accessibility data, such as MNase-seq, provide valuable insights into this landscape and have been used to compute chromatin occupancy profiles. Multiple datasets generated over time or under different conditions can thus be used to study dynamic changes in chromatin occupancy across the genome.

**Results:**

Our existing model, RoboCOP, computes a genome-wide chromatin occupancy profile for nucleosomes and hundreds of transcription factors. Here, we present a new method called DynaCOP that takes multiple chromatin occupancy profiles and uses them to generate a series of nucleosome-guided difference profiles. These profiles identify differentially binding transcription factors and reveal changes in nucleosome occupancy and positioning. We apply DynaCOP to chromatin occupancy profiles derived from deeply sequenced time-series MNase-seq data to study differential chromatin occupancy in the yeast genome under cadmium stress. We find strong correlations between the observed chromatin changes and changes in transcription.

**Availability and implementation:**

https://github.com/HarteminkLab/RoboCOP

## 1 Introduction

The chromatin architecture, or epigenomic landscape, is the arrangement of proteins across the genome. Chromatin is dynamic, and its architecture is constantly changing due to internal and external factors. Changes in chromatin architecture result from, and contribute to, changes in gene expression (Li and Reinberg 2011). Proteins that bind the genome to regulate gene expression include transcription factors (TFs) and histone octamers, which wrap 147 bases of DNA around themselves to form nucleosomes (Kornberg and Lorch 1999). Nucleosomes can occupy different positions along the genome, thereby allowing or blocking the binding of other proteins, including TFs, which activate or repress transcription.

The genomic occupancy of proteins can be studied using antibody-based methods that reveal the binding locations of individual proteins with high precision, but only one at a time (Harbison *et al.* 2004, Rhee and Pugh 2011, Rossi *et al.* 2021). Applying these assays to study the hundreds or thousands of distinct proteins binding to the genome is impractical. In contrast, chromatin accessibility assays use enzymes like MNase (Henikoff *et al.* 2011), DNase (Hesselberth *et al.* 2009), or Tn5 transposase (Buenrostro *et al.* 2013) to cleave accessible DNA, providing an overview of the epigenomic landscape in terms of bound and unbound regions of the genome. Paired-end sequencing of the resulting fragments provides insight into the sizes of proteins bound at individual loci (Tsompana and Buck 2014), especially in the case of MNase due to its exonuclease activity. In conjunction with prior knowledge about sequence specificity and the size of binding sites, the data from a single chromatin accessibility assay can be used to profile the occupancy of hundreds of distinct proteins genome-wide (Zhong *et al.* 2014, Mitra *et al.* 2021).

In previous work, we developed a model called RoboCOP that computes a probabilistic occupancy profile for nucleosomes and TFs (collectively called DNA-binding factors, or DBFs) across the genome using MNase-seq and/or ATAC-seq data, along with the nucleotide sequence (Mitra *et al.* 2021). RoboCOP uses a hidden Markov model (HMM) where each hidden state corresponds to a single base of a TF binding site or any one of the 147 bases of a nucleosome. Thus, the model produces a chromatin occupancy profile at single base-pair resolution.

While RoboCOP provides useful insight into the epigenomic landscape of the genome for a given chromatin accessibility dataset, we still need tools to systematically identify changes in the chromatin architecture over time or under different experimental conditions. Previously developed tools have used epigenomic data to study changes in nucleosome positions for pairs of datasets (Buitrago *et al.* 2019) or have separately annotated the TF binding sites in different datasets (Chen *et al.* 2010, Li *et al.* 2019, Bentsen *et al.* 2020). In this paper, we report an extension of RoboCOP, called DynaCOP (dynamic chromatin occupancy profiler), that simultaneously identifies changes in nucleosome occupancy and TF binding across multiple datasets.

We use a time-series MNase-seq dataset of yeast cadmium treatment (Tran *et al.* 2021) to highlight the utility of DynaCOP. The available MNase-seq data are deeply sequenced, which is helpful for learning accurate chromatin occupancy profiles using RoboCOP. Meanwhile, cadmium treatment of yeast cells is known to generate a stress response that causes genome-wide changes in chromatin accessibility (Tran *et al.* 2021). We therefore consider this dataset a suitable test case for DynaCOP.

We first use DynaCOP to compute a nucleosome map of the entire genome by linking the nucleosomes across all time points of cadmium treatment. Next, we link the TF binding sites across all time points and generate visualizations highlighting the differences. Further analyses reveal distinct groups of nucleosomes based on their changes over time. We also develop and employ a gene-centric approach which allows us to identify clear patterns among nucleosomes associated with individual genes (+1, −1, and gene body nucleosomes) that are linked to changes in gene expression. Finally, we identify groups of TFs that bind differentially in the promoter regions of these genes. To the best of our knowledge, DynaCOP is the first method that utilizes probabilistic chromatin occupancy profiles of the genome to simultaneously profile dynamic changes in nucleosome positions and TF binding sites genome-wide at base-pair resolution.

## 2 Materials and methods

### 2.1 DynaCOP processes RoboCOP output to identify differences in chromatin

The RoboCOP model (Mitra *et al.* 2021) takes three sets of observed data as input: (i) the genomic nucleotide sequence (**s**), (ii) midpoint counts of paired-end MNase-seq/ATAC-seq fragments whose length corresponds to that of a nucleosome (we use fragments of length between 127 and 187, henceforth called nucFrags or **l**), and (iii) midpoint counts of short fragments indicative of TF binding sites (we use fragments of length less than 80, henceforth called shortFrags or **m**). Midpoint counts of the different-length MNase-seq/ATAC-seq fragments are modeled using negative binomial distributions, conditioned on the hidden binding state being a TF, a nucleosome, or unbound. Conditioned on the same hidden state, genomic nucleotides are modeled using a position weight matrix (PWM) if they are bound by a TF, a dinucleotide PWM if they are bound by a nucleosome, or a generic background distribution if they are unbound. As an innovation on our previous version of RoboCOP in which the dinucleotide PWM for nucleosomes was precomputed for the yeast genome (Wasson and Hartemink 2009), here we calculate the dinucleotide PWM using annotated locations of well-positioned nucleosomes if those are provided as input. Nucleosomes are known to have weak sequence specificity that can vary between organisms (Kaplan *et al.* 2009).

After running RoboCOP on multiple datasets, we run DynaCOP to identify differences in the chromatin occupancy profile of the genome across all of the outputs. RoboCOP generates nucleosome predictions separately for individual epigenomic datasets. We link the RoboCOP-predicted nucleosomes across all outputs to create a nucleosome difference map that we describe in the next section. The nucleosome difference map highlights changes in occupancy and positional shifts across all datasets. DynaCOP also links TF binding sites across multiple RoboCOP outputs using an approach described in the subsection below entitled “TF binding site calling and matching.” Once we have linked the nucleosomes and TF binding sites across RoboCOP outputs, we create visualizations plotting the probabilistic occupancy profile of each DBF and highlighting notable differences.

### 2.2 Nucleosome calling and linking

Nucleosomes are called in a greedy manner using the RoboCOP predictions of nucleosome dyads, P(dyad|**s**, **l**, **m**) (Zhong *et al.* 2016, Mitra *et al.* 2021). Briefly, we iteratively select as a nucleosome dyad the genomic location with the highest P(dyad|**s**, **l**, **m**) and remove all locations within a window of 117 bases centered on that dyad from being called as a nucleosome in future rounds (we chose 117 instead of 147 to allow some overlap). Since RoboCOP models each of the 147 positions of the nucleosome as hidden states, we can thus calculate, for every genomic location, the probability of it being occupied by any of the 147 positions within a nucleosome, P(nuc|**s**, **l**, **m**), by summing the posterior of the 147 hidden states of a nucleosome. We use this nucleosome probability to set a threshold of 0.1 when calling nucleosome positions from RoboCOP output.

After calling the nucleosome positions separately for all RoboCOP outputs, we link the nucleosomes using DynaCOP. We first link nucleosomes from two of the outputs. When we are using MNase-seq from a time-series experiment, we start by linking the nucleosome positions from the first and second time points. For each chromosome, we scan the genome coordinates from beginning to end. If a pair of nucleosomes in the two sets are at most half a nucleosome (73 bases) apart, then the two nucleosomes are linked together. If a nucleosome cannot be linked to any nucleosome in the other dataset, it is added as a new nucleosome in the linkage map. After linking the nucleosomes from the two outputs, we calculate how much each nucleosome has shifted. In the context of a time series, nucleosome predictions for later time points are incorporated in the same manner, iteratively: We link the nucleosome predictions from the third time point to the linkage map created using the first two time points and continue linking the nucleosome predictions from later time points in a similar manner. Note that nucleosome shifts are only computed for consecutively added sets of nucleosomes. Upon creating this nucleosome linkage map, we can use it to study how the occupancy profile of each individual nucleosome changes over time or across conditions.

### 2.3 Nucleosome shift categorization

We use the nucleosome linkage map to categorize nucleosomes into four distinct classes. The first is when a nucleosome is not present across all times, in which case it is categorized as “not always present.” When a nucleosome is present across all times, we check whether it remains at the same position across all datasets, in which case it is categorized as “no shift.” We find that the aggregated nucleosome shift signal has a 10 base-pair periodicity that potentially arises from the periodic nature of the weak sequence specificity of nucleosomes (Supplementary Fig. S1) (Mitra *et al.* 2021). Additionally, a nucleosome shift of approximately 20 bases corresponds to 1.8 times the standard deviation of the shift distribution. Thus, to accommodate some positional noise, we consider a nucleosome to have remained at the same position if it does not shift by more than 20 bases left or right. If, however, a nucleosome undergoes an absolute shift of more than 20 bases, and if all such large shifts between consecutive time points for that nucleosome are either positive or negative, it is categorized as a case of “directional shift.” Finally, if a nucleosome undergoes an absolute shift of more than 20 bases but such large shifts are a mix of positive and negative values, then it is considered to have exhibited “nondirectional shift.” Note that in cases with only two sets of RoboCOP outputs, nucleosomes with “nondirectional shift” cannot exist. In Supplementary Table S1, we provide an example table of annotations of nucleosome linkage and shifts.

### 2.4 Nucleosome occupancy clustering

The probability of the nucleosome dyad, P(dyad|**s**, **l**, **m**), computed by RoboCOP provides an estimate of how well-positioned or fuzzy a nucleosome is (Supplementary Fig. S3). A nucleosome is fuzzy if the nucleosome dyad is less well-positioned in the cell population and therefore the probability of the dyad, P(dyad|**s**, **l**, **m**), is spread out over multiple adjacent positions, leading to an overall lower P(dyad|**s**, **l**, **m**) compared to that of a well-positioned nucleosome. The fuzziness of a nucleosome can change under different experimental conditions. To study this change in nucleosome fuzziness, we use DynaCOP to cluster the previously linked nucleosomes based on fuzziness. Each linked nucleosome is associated with a nucleosome dyad probability, P(dyad|**s**, **l**, **m**), for every linked dataset. If the linked nucleosome is missing in at least one dataset (“not always present”), we assign a dyad probability of 0. We cluster the nucleosome dyad probabilities of the linked nucleosomes using *k*-means clustering with Euclidean distance (Pedregosa *et al*. 2011).

### 2.5 Nucleosome labeling with respect to reference annotations

We use the nucleosome annotations of Chereji *et al.* (2018) to assign +1 and −1 labels to a subset of the nucleosomes. The following labels are assigned in decreasing order of preference: (i) +1 nucleosome or −1 nucleosome if the linked nucleosome is at most 50 bases from annotated +1 and −1 nucleosomes, (ii) promoter nucleosome if the nucleosome is present between +1 and −1 nucleosomes, (iii) gene body nucleosome if the nucleosome is present within the transcript or open reading frame (ORF), whichever is longer, and (iv) intergenic nucleosome if it is not present in any of the other categories.

### 2.6 Gene-centric analysis

For the gene-centric analysis, the +1, −1, and gene body nucleosomes are selected for each gene for clustering. In this analysis, we are using a time-series experiment, so we calculate nucleosome shifts with respect to nucleosome positions in the first time point. The nucleosome shifts of the +1 and −1 nucleosomes are taken as features (the sign is flipped if the gene lies on the Crick strand). Additional features include the dyad probability, P(dyad|**s**, **l**, **m**), of +1 and −1 nucleosomes and the average dyad probability of all nucleosomes within the gene body. The five features are then standardized, followed by *k*-means clustering with Euclidean distance (Pedregosa *et al*. 2011).

### 2.7 TF binding site calling and matching

TF binding sites are first called separately for each output of RoboCOP. We consider a site to be bound by a TF if P(TF|**s**, **l**, **m**) ≥ 0.1. Unlike nucleosomes, TF binding sites do not shift along the genome; the TF binding sites are sequence-specific, so the location of a TF binding site remains the same across all RoboCOP outputs. Therefore, linking TF binding sites across multiple RoboCOP outputs is straightforward. We consider a site to be differentially bound by a TF across the samples if the minimum binding probability is less than 0.1 and the maximum binding probability is greater than 0.1 at the same site.

### 2.8 Time-series data from yeast cells exposed to cadmium

In this article, we demonstrate the utility of DynaCOP using genomic MNase-seq data collected from a population of yeast cells undergoing cadmium treatment (Tran *et al.* 2021). We apply DynaCOP to the chromatin occupancy profiles learned from MNase-seq datasets collected at 0, 7.5, 15, 30, and 60 min following cadmium treatment. We interpret our findings by comparing the changes therein to changes in gene expression using RNA-seq datasets that were collected at the same time points (Tran *et al.* 2021).

## 3 Results

### 3.1 DynaCOP creates visualizations highlighting differences in chromatin architecture

**Figure 1.**
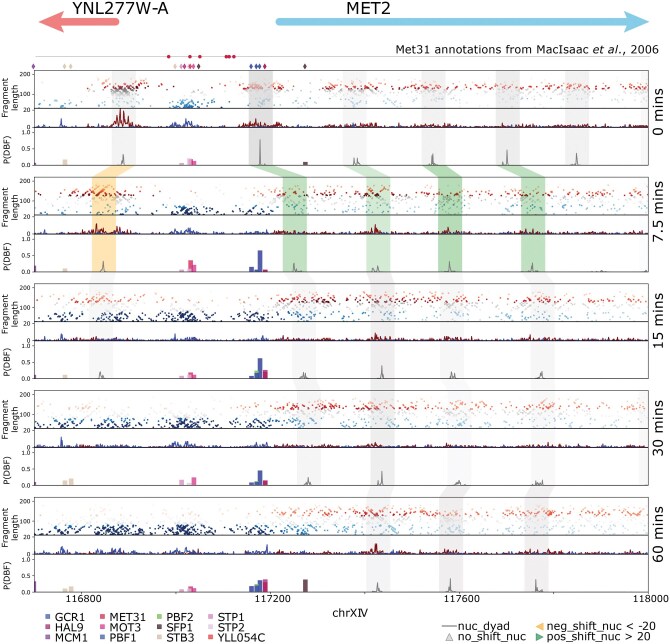
DynaCOP output plot highlights chromatin changes during cadmium treatment in MET2 promoter region (chrXIV:116700–118000) at 0, 7.5, 15, 30, and 60 min. The five panels corresponding to those five times each contain three plots. The topmost plot of each panel depicts the midpoints of MNase-seq fragments for every genomic position (*x*-axis) and fragment length (*y*-axis). Fragments of length 127–187 (nucFrags, colored red) are used to predict nucleosome positions; fragments of length <80 (shortFrags, colored blue) are used to predict TF binding sites. The middle plot of each panel is a flattened 1-D signal of the aggregate midpoint counts of nucFrags (red) and shortFrags (blue). RoboCOP is run separately on each of the five MNase-seq datasets. The probability of the nucleosome dyad P(dyad|**s**, **l**, **m**) as predicted by RoboCOP is plotted using a gray curve in the bottom plot of each panel. The predicted dyads frequently align with accumulated 1-D nucFrags signal. DynaCOP links RoboCOP-predicted nucleosomes across time using bands (gray, orange, and green) that span 51 bases centered on the predicted dyads. The opacity of the bands is proportional to the predicted probability of the dyad. The band is green if the nucleosome shifts right (i.e. downstream on the Watson strand) by more than 20 bases compared to the previous time point, orange if it shifts left by more than 20 bases, and gray otherwise. TFs are considered to bind differentially if the maximum binding probability at the given location exceeds 0.1 and the minimum binding probability is less than 0.1 at the same site. Diamonds above the top of the first panel (below the gene annotations) depict the sites of all bound TFs (whether binding differentially or not). Circles above the diamonds depict annotated potential Met31 binding sites (MacIsaac *et al.* 2006). The legend at the bottom lists the colors used for the TF binding site predictions, P(TF|**s**, **l**, **m**).

DynaCOP processes the RoboCOP output from multiple datasets to link nucleosomes, reveal changes in nucleosome occupancy and positioning, and identify dynamic binding sites of several TFs. DynaCOP creates visualizations highlighting these differences in chromatin occupancy profiles. In Fig. 1, we show the DynaCOP output for the MET2 promoter across 60 min of cadmium treatment. For each time point, we plot the two-dimensional MNase-seq signal followed by the flattened 1-D signal of nucFrags in red and shortFrags in blue. These flattened 1-D signals are used as input for RoboCOP. The RoboCOP outputs are then processed by DynaCOP and visualized in each time point’s third plot. In that plot, nucleosome dyad predictions (P(dyad|**s**, **l**, **m**)) are shown using a gray curve. The dyad predictions have higher probabilities for well-positioned nucleosomes and lower probabilities for fuzzy nucleosomes. We plot the computed nucleosome linkages using gray, orange, and green bands spanning 51 bases centered on the predicted nucleosome dyads in Fig. 1. The band is drawn in orange if the nucleosome at the later time shifts more than 20 bases left (i.e. upstream on the Watson strand). The band is green if the nucleosome at the later time shifts more than 20 bases right (i.e. downstream on the Watson strand). The band is gray otherwise. These bands are drawn with a level of saturation proportional to the nucleosome prediction P(dyad|**s**, **l**, **m**) at that time point. A nucleosome predicted with high probability is more well-positioned and is drawn with a more opaque band; in contrast, a nucleosome predicted with low probability is drawn with more transparency. At times, nucleosomes are evicted or otherwise not present in all time points. In such cases, the linkage is disconnected. Additionally, we find that predicted nucleosomes mostly line up with the enrichment of nucleosomal fragments in MNase-seq; however, in certain instances the predictions appear to be slightly shifted from the MNase-seq enrichment signal. This occurs for two reasons. First, RoboCOP models the nucleosomal signal using both shape and count parameters, which might allow the predictions to shift slightly to accommodate the shape of the signal. Second, RoboCOP assumes all DBFs are competing to bind to the genome, which means that the learned nucleosome positions can adjust to accommodate the learned positions of TFs or other nucleosomes nearby. We have previously shown in Mitra *et al.* (2021) that incorporating this competitive binding yields more accurate nucleosome predictions compared to methods that derive nucleosome occupancy solely from peak MNase-seq signals.

In addition to nucleosome differences, the plot also highlights the binding dynamics of TFs. TF binding sites that are predicted by RoboCOP with a probability P(TF|**s**, **l**, **m**) ≥0.1 in at least one time point are depicted using diamonds below the gene annotations in Fig. 1. If a TF binds differentially, then its probabilistic predictions for each time are plotted in the DynaCOP output panels in Fig. 1. This enables us to visualize differences and at the same time be aware of all the binding site predictions, differential or otherwise, as indicated by the diamonds. In Fig. 1, RoboCOP predicts the differential occupancy of one of the annotated binding sites of Met31 (MacIsaac *et al.* 2006, Rossi *et al.* 2021), a key regulator of MET2.

MET2 is involved in methionine biosynthesis and is part of the sulfur pathway that is up-regulated during cadmium stress (Tran *et al.* 2021). We find that the chromatin architecture around MET2 undergoes noticeable changes during cadmium stress. The −1 nucleosome that forms the upstream promoter boundary of the nucleosome-free region (NFR) for MET2 progressively moves upstream during the course of cadmium stress, shifting upstream by 43 bases within 7.5 min. The +1 nucleosome further opens up the NFR by shifting downstream, notably by 72 bases within 7.5 min. Additionally, the +2 and +3 nucleosomes shift downstream, allowing the +1 nucleosome enough space to move downstream. The −1 nucleosome is lost at 30 and 60 min, most likely because the nucleosome signal becomes so fuzzy—possibly due to the movement of Pol II at the gene promoter. Interestingly, we find that as the +1 nucleosome moves downstream after 7.5 min of cadmium treatment, RoboCOP predicts TF binding sites within the newly expanded NFR. One of the TFs is Gcr1 (depicted in blue), a glycolytic activator associated with stress response genes (Tran *et al.* 2021).

This example illustrates DynaCOP’s ability to generate plots that visualize key changes in the chromatin architecture over the course of an experiment.

### 3.2 Categories of nucleosome shift

**Figure 2.**
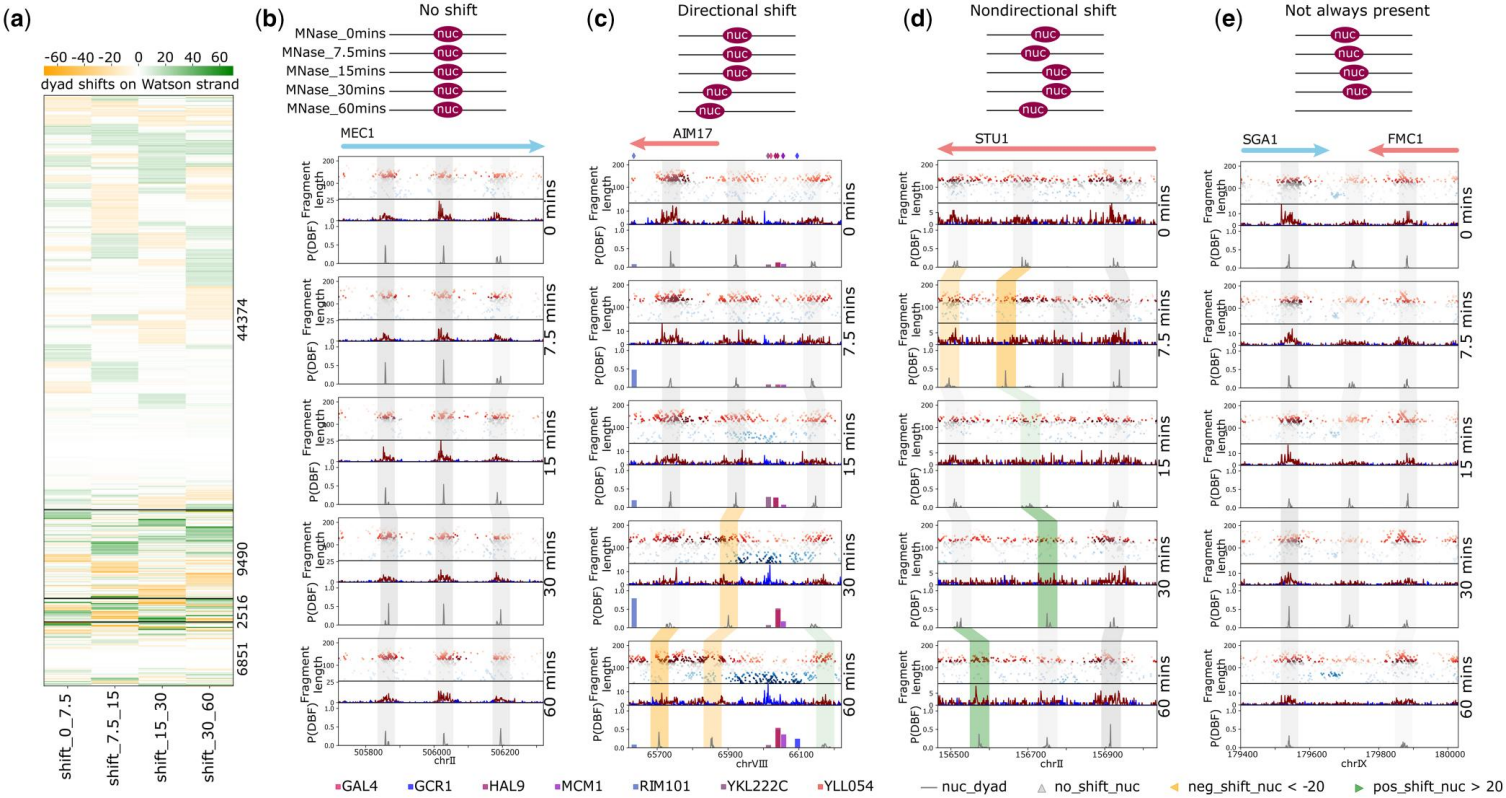
DynaCOP links nucleosomes across all time points of cadmium treatment and calculates the nucleosome shifts between pairs of consecutive time points. (a) Heatmap of the nucleosome shifts grouped by the four categories from top to bottom: no shift (44374 nucleosomes), directional shift (9490 nucleosomes), nondirectional shift (2516 nucleosomes), and not always present (6851 nucleosomes). (b–e) Plots are centered on the example nucleosomes. (b) The nucleosomes are categorized as no shift if the nucleosome does not shift by more than 20 bases (upstream or downstream) across all times. (c) The nucleosome is considered to undergo directional shift if the calculated pairwise shifts are ever greater than 20 bases between consecutive times and are either all upstream or all downstream. (d) If the shifts greater than 20 bases are not all in the same direction, the nucleosome is categorized as having nondirectional shift. (e) If the nucleosome is not detected at all time points, then it is labeled as not always present.

In addition to generating visualizations that help us understand the dynamics of chromatin occupancy at specific loci, we can use DynaCOP to analyze the patterns of changes across the entire genome. One such pattern is nucleosome shift dynamics. We categorized the linked nucleosomes from the cadmium time series into four groups. The vast majority (44374 nucleosomes) have “no shift” (Fig. 2a and b), as studies indicate that cadmium stress causes chromatin changes only at specific regions of the genome. A sizable fraction (9490 nucleosomes) exhibit “directional shift,” where the shift occurs in a consistent direction across all time points. For example, in Fig. 2c, the nucleosome at the start of the AIM17 gene progressively shifts downstream, opening up space in the promoter. We validated using available RNA-seq data (Tran *et al.* 2021) that the expression of AIM17 increases monotonically with cadmium treatment (Supplementary Fig. S2), uncovering a clear connection between nucleosome shifts and transcription in this instance.

Nucleosomes can alternatively show “nondirectional shift,” meaning their movement is not consistent in a single direction. In our data, only a small fraction (2516 nucleosomes, or 3%) of nucleosomes fall in this category (Fig. 2a). Fig. 2d shows an example of a nucleosome shifting nondirectionally within the gene body of STU1. Such shifts might be of particular interest when the data support periodic dynamics, as would be the case for cell cycle experiments (Nocetti and Whitehouse 2016). Finally, 10% of nucleosomes are categorized as “not always present” (6851 nucleosomes) (Fig. 2a). This can happen when a nucleosome is evicted (or appears) during the course of an experiment, or becomes partially unwrapped due to high transcription and the resulting MNase-seq signal contains fragments much shorter than typical for nucleosomes. In Fig. 2e, we see a nucleosome that has been lost near the transcription termination site of SGA1 and FMC1. Examining the transcription of these two genes, we find that both are up-regulated upon cadmium treatment, particularly SGA1 (Supplementary Fig. S2).

### 3.3 Clusters of nucleosome occupancy

Each nucleosome predicted by RoboCOP has a fuzziness score defined by the posterior probability of the nucleosome dyad, P(dyad|**s**, **l**, **m**). A higher posterior indicates a more well-positioned nucleosome (Supplementary Fig. S3). A nucleosome is considered well-positioned when the nucleosome occupies the same position across the cell population, which concentrates its probability at a single location. In contrast, a fuzzy nucleosome exhibits positional variation, lowering its probability at any one position. Our model also predicts a lower probability for a nucleosome when it is competing with TFs to occupy a given location.

**Figure 3.**
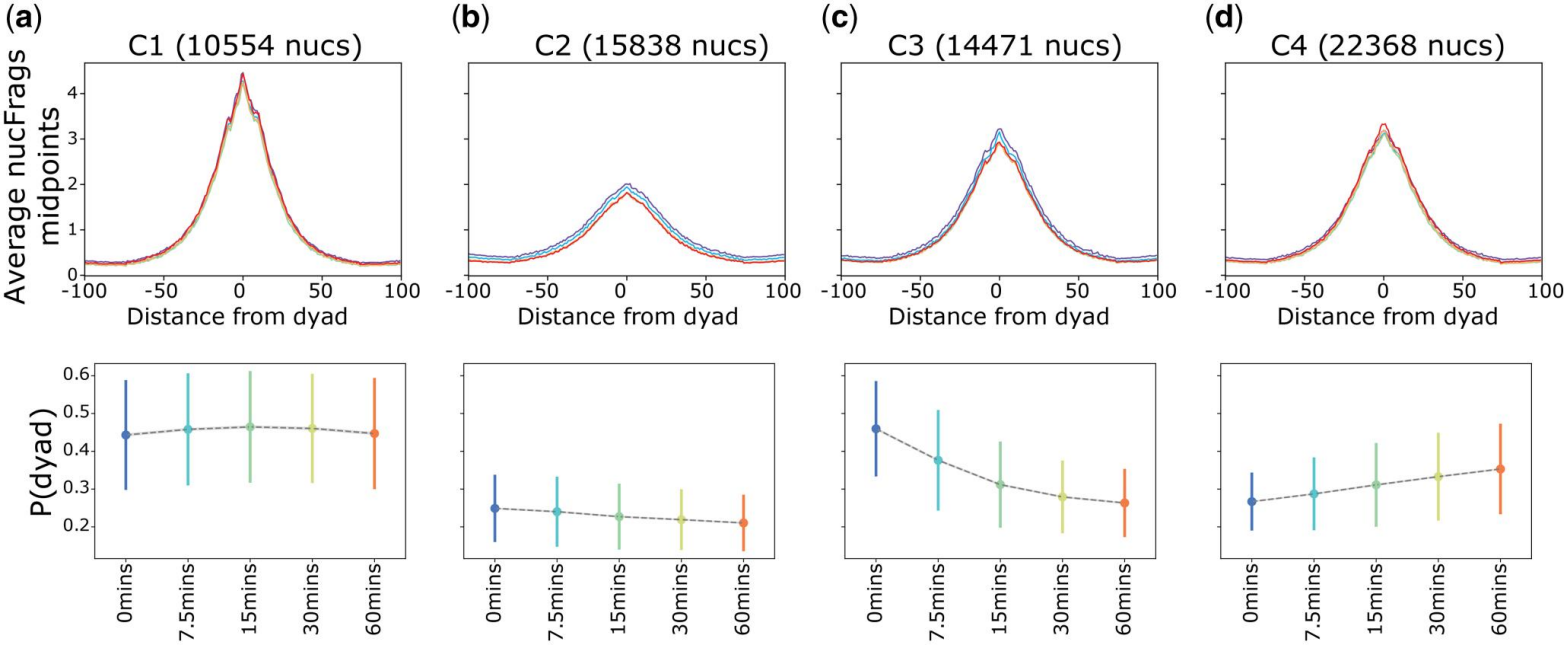
DynaCOP clusters the nucleosome dyad posterior, P(dyad|**s**, **l**, **m**), for nucleosomes linked across cadmium treatment. The top panel shows the MNase-seq midpoint counts of nucFrags at all time points, and the bottom panel shows the mean P(dyad|**s**, **l**, **m**) for each time point, along with the standard deviation. The four clusters show nucleosomes that are (a) well-positioned across all time points, (b) not well-positioned across all time points and become slightly fuzzier with time, (c) well-positioned at the beginning but become fuzzier with time, and (d) initially fuzzy but become more well-positioned with time.

In our cadmium dataset, we cluster nucleosome occupancy profiles across the five time points and obtain four distinct clusters, each showing distinct changes in fuzziness over time. Cluster C1 (10554 nucleosomes) is comprised of the most well-positioned nucleosomes, predicted with high probability across all time points (bottom of Fig. 3a). The distribution of nucFrags around the dyads of nucleosomes in cluster C1 likewise remains the same over the entire time course (top of Fig. 3a). Cluster C2 (15838 nucleosomes) consists of nucleosomes that are not well-positioned at the start of the time course and become slightly fuzzier over time (Fig. 3b). This cluster contains roughly one-fourth of the total number of yeast nucleosomes. Another quarter of the nucleosomes are in cluster C3 (14471 nucleosomes). These nucleosomes begin as well-positioned but become ever fuzzier over time (Fig. 3c). Nucleosomes of this kind are expected in our data because highly transcribed genes (such as those responding to cadmium stress) tend to have more disorganized nucleosomes due to the polymerase transcribing the gene (Tran *et al.* 2021). We notice the opposite phenomenon in cluster C4 (22368 nucleosomes), where the nucleosomes start off being less well-positioned but gradually become more so (Fig. 3d). We expect such nucleosomes in genes whose transcription decreases over the time course (Tran *et al.* 2021).

### 3.4 Relation between nucleosome shift and occupancy

**Figure 4.**
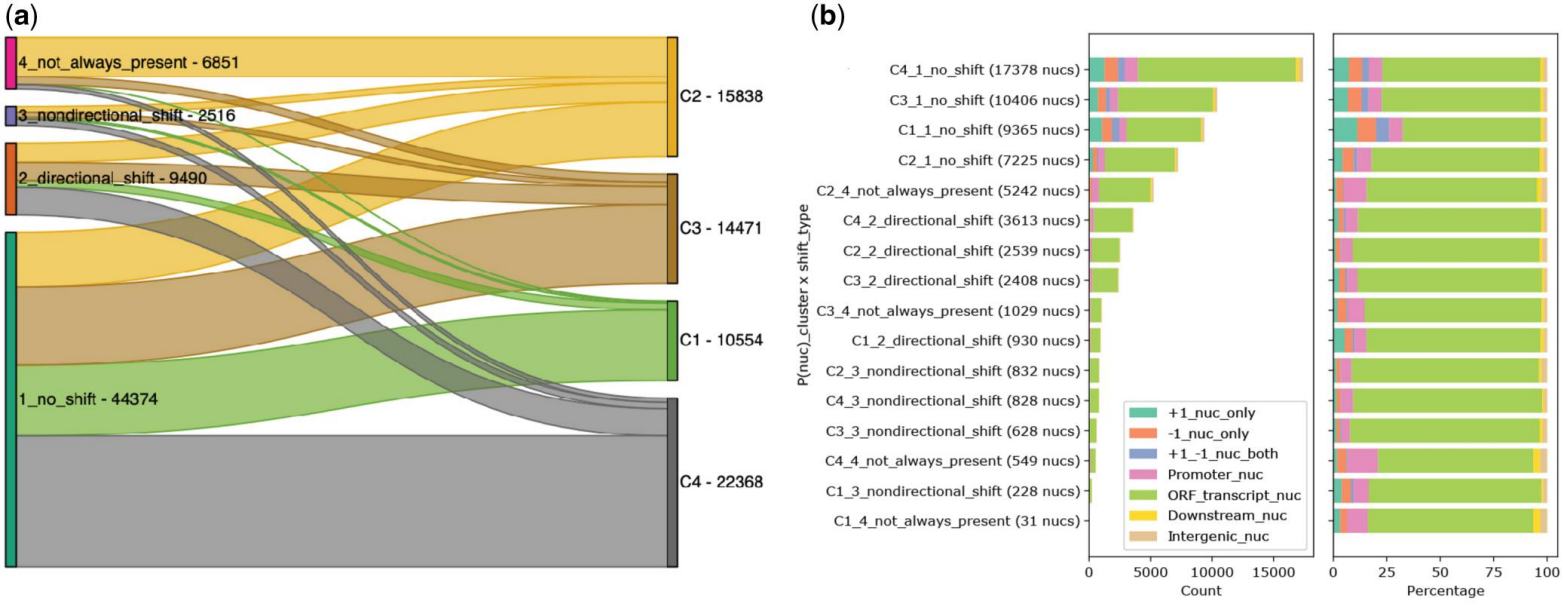
(a) Flow diagram showing the relationship between shift types and fuzziness clusters of nucleosomes. (b) Bar plots showing the distribution of the nucleosomes categorized by shift and fuzziness.

Having identified the types of shifts and profiles of occupancy exhibited by nucleosomes over the 60 min of cadmium treatment, we compared the shift types to the occupancy profiles (Fig. 4a). We find that shift types and occupancy profile clusters do not share a one-to-one relationship. The nucleosomes that do not shift can be found in any of the four clusters of occupancy profiles, C1–C4. This means that while the nucleosome position remains the same, the fuzziness of these nucleosomes may change in various ways over the time course. Furthermore, these four categories of “no shift” nucleosomes (C1_no_shift, C2_no_shift, C3_no_shift, and C4_no_shift) form the four largest categories of nucleosomes (Fig. 4a). Almost all the nucleosomes in cluster C1 do not shift over the time course (C1_no_shift: 9365 nucleosomes) and are less abundant in gene bodies (Fig. 4b). In contrast, a subset of the nucleosomes in cluster C2 that do not shift over time (C2_no_shift: 7225 nucleosomes) are more enriched in gene bodies. The nucleosomes with directional shift are split approximately evenly between clusters C2, C3, and C4 (Fig. 4a). Nucleosomes that are not always present are mainly fuzzy nucleosomes in cluster C2 (5242 nucleosomes). Interestingly, these nucleosomes are rarely labeled as +1 nucleosomes (Fig. 4b). This makes sense because +1 nucleosomes are generally considered to be well-positioned. Given the nature of our data, very few nucleosomes exhibit nondirectional shift, and these are mainly split among clusters C2, C3, and C4.

This analysis highlights how nucleosome properties of shifts and occupancy profiles are interconnected. It also highlights how these properties are related to the genomic context of the nucleosome positions.

### 3.5 Gene-centric chromatin dynamics

**Figure 5.**
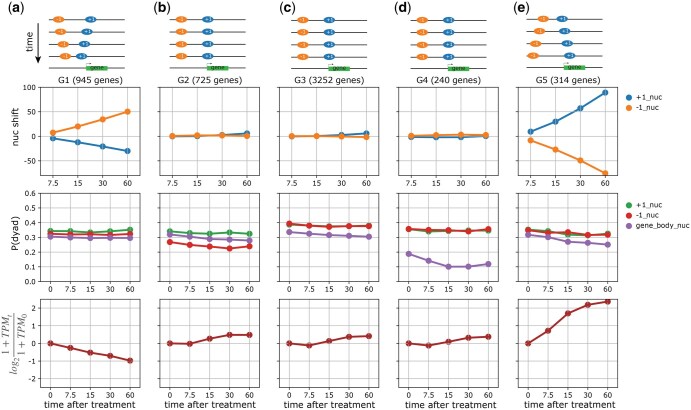
(a–e) Gene-centric cluster analysis of DynaCOP-linked nucleosomes using nucleosome shifts of the +1 and −1 nucleosomes (blue and orange curves in the top panel and the schematic above the panels), as well as the probability of the nucleosome dyad, P(dyad|**s**, **l**, **m**), for +1, −1, and average gene body nucleosomes (green, red, and purple curves in the middle panel). Nucleosome shifts are with respect to the first time point (0 min). Cluster-wise mean of log-fold change in RNA-seq of matched time points (not used in clustering) is plotted in brown in the bottom panel to allow comparison of dynamics of chromatin architecture and transcription. Here, *x*-axes denote the time points of the cadmium treatment experiment.

In the previous analysis, we observed that nucleosome profiles have distinct characteristics with respect to gene bodies. We therefore decided to use gene annotations to group these nucleosomes in a gene-centric way to see whether we could identify patterns that relate to gene expression dynamics. To this end, for each gene, we used the positional shifts and the occupancy profiles, P(dyad|**s**, **l**, **m**), of its +1 and −1 nucleosomes, as well as the average occupancy profile of the nucleosomes present within its gene body. Using these features, we employed *k*-means clustering with five clusters and identified unique chromatin architectures for each cluster (Fig. 5). Then, using available RNA-seq data for matched time points, we assessed the nucleosome dynamics of the five clusters and their relation to gene expression. Note that we used the gene expression data only for assessment; the clustering was performed solely on the nucleosome features.

Cluster G1 comprises 945 genes that exhibit an upstream shift of the +1 nucleosome (blue curve in Fig. 5a) and downstream shift of the −1 nucleosome (orange curve in Fig. 5a) during cadmium treatment, resulting in a closing of the NFR. Examining available gene expression data, we find that these genes are down-regulated over the course of the treatment (brown curve in Fig. 5a). Unsurprisingly, gene ontology (GO) enrichment analysis highlights that the genes are primarily ribosomal (Supplementary Fig. S4). Ribosomal genes are known to be down-regulated during cadmium stress, and the nucleosomes become more organized under such conditions (Tran *et al.* 2021). Cluster G3 contains a majority of the genes and is also enriched for ribosomal genes, along with cell cycle genes (Supplementary Fig. S4). However, these genes do not exhibit notable chromatin or transcriptional dynamics (Fig. 5c). The genes in cluster G2 are associated with the plasma membrane and likewise do not show chromatin or transcriptional dynamics, even though the associated nucleosomes have a distinct chromatin architecture. Likewise, the genes related to cluster G4 do not show any gene expression dynamics, although the gene body nucleosomes are not well-positioned and become progressively less so over time. This cluster is enriched with mitochondrial genes. Finally, the +1 and −1 nucleosomes of the genes in cluster G5 show remarkable shifts over time that are opposite to the dynamics of those in cluster G1. The NFRs in the promoter regions of the genes are expanded by shifting the +1 nucleosome downstream and the −1 nucleosome upstream. We find that these genes show increased expression during cadmium treatment (Fig. 5e) and are linked to stress response pathways (Supplementary Fig. S4) known to be up-regulated during cadmium treatment (Tran *et al.* 2021).

The gene-centric analysis highlights the distinct chromatin architectures of different clusters of genes. Some of the chromatin architectures reflect temporal changes that relate to transcriptional dynamics (clusters G1 and G5). We also find clusters with distinct chromatin architectures that do not exhibit marked changes over time but are still enriched for particular GO terms.

### 3.6 TF activity in gene clusters

Next, we wanted to identify the TFs that putatively regulate the chromatin dynamics of the identified clusters and compare these regulations to gene expression (RNA-seq) for matched time points. Here we focus on differential binding sites that lie within the promoter regions. To find cluster-specific TF enrichment, we retain the TFs that bind differentially to at least 2% of the gene promoters within a cluster.

**Figure 6.**
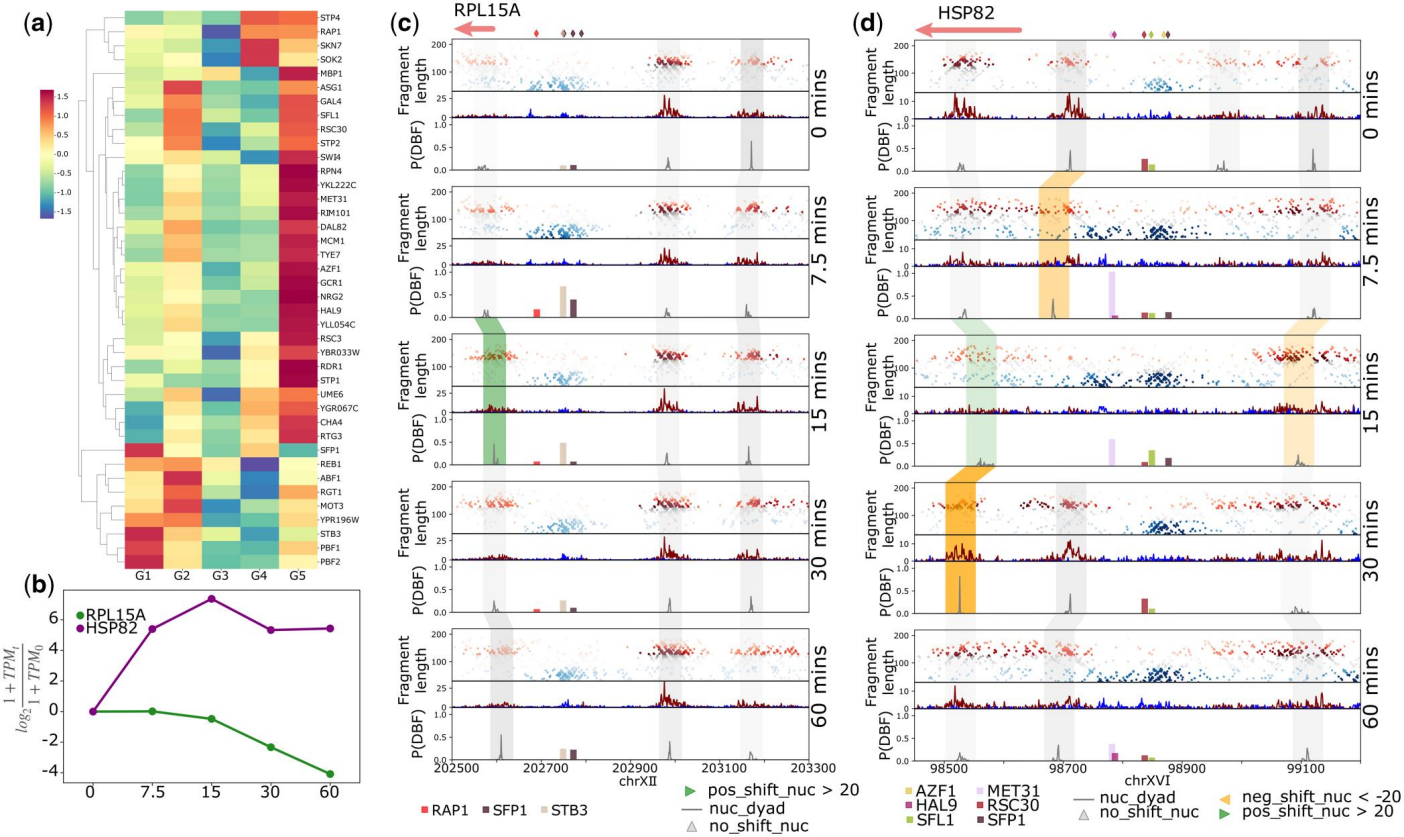
(a) Heatmap of percentage of gene promoters that each TF differentially binds during cadmium treatment. Percentages are transformed to *z*-scores. Selected TFs differentially bind to at least 2% of genes in one cluster for clusters identified in Fig. 5. (b) Gene expression (RNA-seq) of RPL15A (present in cluster G1) and HSP82 (present in cluster G5) at 0, 7.5, 15, 30, and 60 min after cadmium treatment. (c) Rap1 binding in the RPL15A promoter decreases during cadmium treatment, and RPL15A expression in (b) is also downregulated. (d) Met31 arrives to bind the HSP82 promoter at 7.5 and 15 min, but is lost at 30 min, and mildly regained at 60 min. The log-fold change in transcription of HSP82 in (b) reflects the same trend.

In our cadmium data, we find differentially enriched TFs are often present in clusters G1 and G5 (Fig. 6a). These two clusters showed temporal changes in chromatin as well as in transcription (Fig. 5). We also find that certain TFs show shared enrichment between pairs of clusters.

As an example from cluster G1, consider RPL15A, a ribosomal protein gene whose transcription is down-regulated over time (Fig. 6b). Rap1 is a known regulator of ribosomal genes (Lieb *et al.* 2001). In Fig. 6c, the level of Rap1 binding in the promoter region of RPL15A decreases over time, permitting the +1 nucleosome to move upstream and reducing the size of the NFR, especially after 15 min. The steady decrease in the transcript level of RPL15A, particularly after 15 min (Fig. 6b), could be related to the decreased level of Rap1 binding.

We also looked at HSP82, which is up-regulated during cadmium stress (Fig. 6b). In Fig. 6d, we find the +1 nucleosome of HSP82 moves downstream, opening up the NFR. In addition, the −1 nucleosome becomes too fuzzy to predict at 7.5 min. Also at 7.5 min, we find Met31, a key regulator of the sulfur pathway (Blaiseau *et al.* 1997), is predicted to bind to the promoter. RoboCOP continues to predict the binding of Met31 at 15 min when the +1 nucleosome is lost. Interestingly, at 30 min we again find signal at the +1 nucleosome that is well-positioned, and Met31 is no longer bound. We also see a decrease in the level of transcription at 30 min in Fig. 6b. At 60 min, the +1 nucleosome has shifted further downstream just enough to see an enrichment of subnucleosomal fragments that allow RoboCOP to again predict mild binding of Met31. We notice the transcription of HSP82 starts to slightly increase again at 60 min.

Through these two examples, we highlight the power of DynaCOP to visualize the dynamics of TF binding and nucleosome positioning and occupancy. Using RNA-seq and MNase-seq from matched time points, we show these chromatin dynamics are in strong agreement with changes in transcription.

## 4 Conclusion

Understanding changes in the epigenomic landscape provides insights into gene regulation. While numerous methods exist to infer the occupancy profiles of individual DBFs (nucleosomes or TFs), we need systematic approaches to compare predictions for multiple DBFs across different conditions to understand the dynamic nature of the chromatin. Here, we develop and apply DynaCOP to study chromatin accessibility changes in yeast cells subjected to cadmium treatment over a time course. We show that DynaCOP elucidates the dynamic occupancy of nucleosome positions and TF binding sites. While our study focuses on a short time series, where most chromatin changes follow a linear pattern, other settings may involve more complex, non-linear gene regulatory changes of subtler biological significance. We hypothesize that under such conditions, DynaCOP will detect a greater number of “nondirectional” nucleosome shifts, which could then be clustered to identify specific subtler patterns.

Additionally, we show that DynaCOP can be used as a visualization tool highlighting the key chromatin occupancy changes in a given genomic locus. Upon clustering the dynamics of the +1, −1, and gene body nucleosomes, we find that DynaCOP identifies distinct groups associated with genes that are up/down-regulated. These genes also show differences in TF binding in the promoter regions, revealing potential cis-regulatory elements.

We have previously demonstrated the robustness of RoboCOP in the predictions made using MNase-seq and/or ATAC-seq (Mitra *et al.* 2021). Our analysis revealed substantial overlaps between predictions made using these two distinct assays, highlighting the reliability of RoboCOP. Furthermore, the findings in this study are in strong concordance with previous findings related to cadmium treatment (Tran *et al.* 2021), reinforcing the utility of DynaCOP. The main limitation of DynaCOP is that it relies on deeply sequenced paired-end chromatin accessibility data, which is expensive to acquire for organisms with longer genome sequences. Due to this limitation, we were only able to apply DynaCOP in the context of yeast, which restricted our ability to assess the generalizability of the tool. In future work, with the availability of more deeply sequenced MNase-seq datasets, we plan to apply DynaCOP to study chromatin dynamics across other systems. Ultimately, we aim to extend this framework to enhance DynaCOP’s versatility, enabling it to analyze chromatin dynamics in various organisms under multiple conditions.

## Supplementary Material

vbaf080_Supplementary_Data

## Data Availability

DynaCOP uses publicly available MNase-seq and RNA-seq data downloaded from GEO under accession number GSE153609.
