# Supplementary material for: Inferring differential protein binding from time-series chromatin accessibility data
Source: Bioinform Adv. 2025 Apr 10;5(1):vbaf080. doi: 10.1093/bioadv/vbaf080 (PMC12037103; doi:10.1093/bioadv/vbaf080)
Supplement: vbaf080_Supplementary_Data [file vbaf080_supplementary_data.pdf]

Supplementary Materials for "Inferring differential protein binding from time-series chromatin accessibility data"

name	chr	dyadA	dyadB	dyadC	scoreA	scoreB	scoreC	shiftAB	shiftBC	shift_type
nuc_1	chrI	1000	1001	998	0.8	0.9	0.3	1	-3	no shift
nuc_2	chrI	2000	2010	2034	0.4	0.5	0.4	10	24	directional shift
nuc_3	chrII	1000	979	1001	0.8	0.9	0.9	-21	22	nondirectional shift
nuc_4	chrIII	3000	2079	2081	0.2	0.7	0.6	-21	2	directional shift
nuc_5	chrX	4000	4001	N/A	0.9	0.4	0.0	1	N/A	not always present
nuc_6	chrXI	N/A	800	N/A	0.0	0.5	0.0	N/A	N/A	not always present

Supplementary Table S1. Example table explaining DynaCOP nucleosome linkage. DynaCOP generated table of nucleosomes linked across multiple RoboCOP outputs using chromatin accessibility data derived from different experimental conditions. Each row corresponds to a nucleosome. Here, each nucleosome has three positions from three different experimental setups. The positions are given by columns dyadA, dyadB, and dyadC within a chromosome chr. The probabilistic prediction of the nucleosome dyad, $P(\text{dyad}|\mathbf{s},\mathbf{l},\mathbf{m})$, as calculated by RoboCOP are given by scoreA, scoreB, and scoreC. Nucleosome shift is calculated by taking the difference between the two dyads from consecutive experiments. So, shiftAB is the difference between dyadA and dyadB. The nucleosome shifts are colored in green if the shift is more than 20 bases, orange if the shift is less than -20 bases, and gray otherwise. If the magnitude of all shifts for a nucleosome is less than 20 bases then the nucleosome undergoes no shift (nuc_1). If all the shifts for a nucleosome with a magnitude greater than 20 are either positive (green) or negative (orange) then the nucleosome undergoes directional shift (nuc_2, nuc_4). If the shifts with magnitude more than 20 are both positive (green) and negative (orange) for two instances then the nucleosome undergoes nondirectional shift (nuc_3). If the nucleosome is not present across all experiments then the nucleosome is not always present (nuc_5, nuc_6). In this case the shift cannot be calculated and is written as N/A in the table.

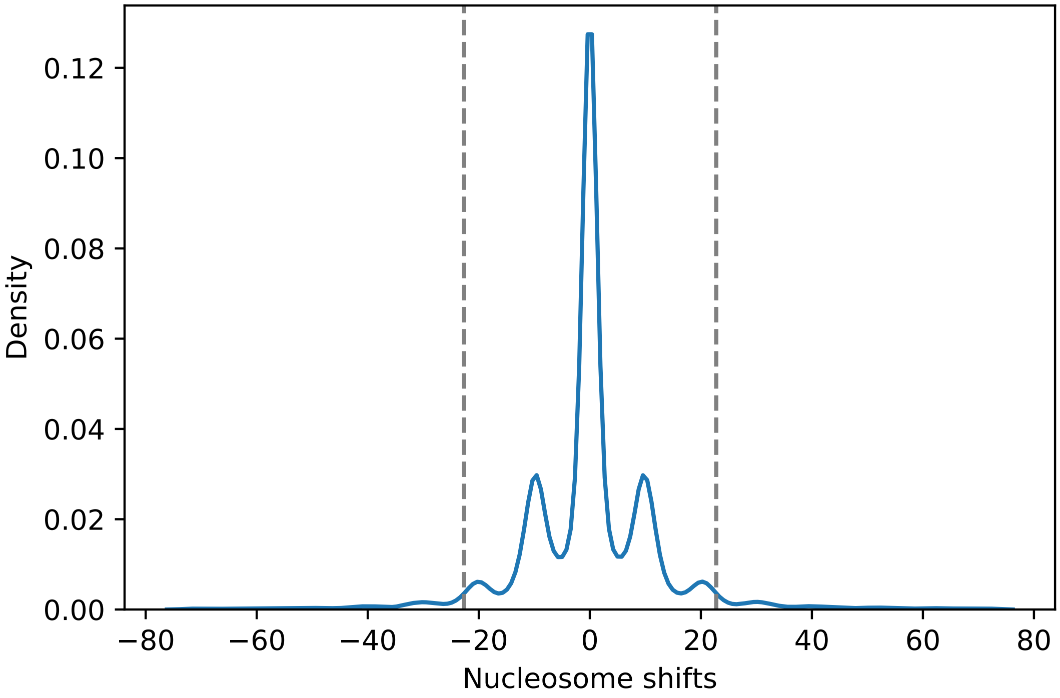

Supplementary Figure S1. Distribution of nucleosome shifts between two consecutive time points. The grey bars denote 1.8 times the standard deviation of the mean shift.

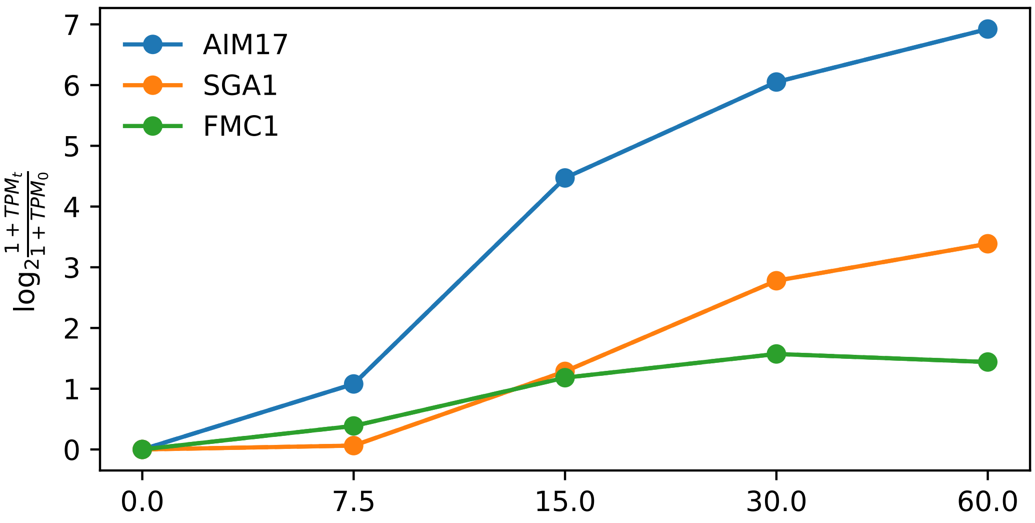

Supplementary Figure S2. Log2 fold change in TPM with respect to TPM at 0 mins of cadmium treatment for genes AIM17, SGA1, and FMC1. All three genes, particularly AIM17 and SGA1, are up-regulated during cadmium treatment. RNA-seq from Tran *et al.*, 2021

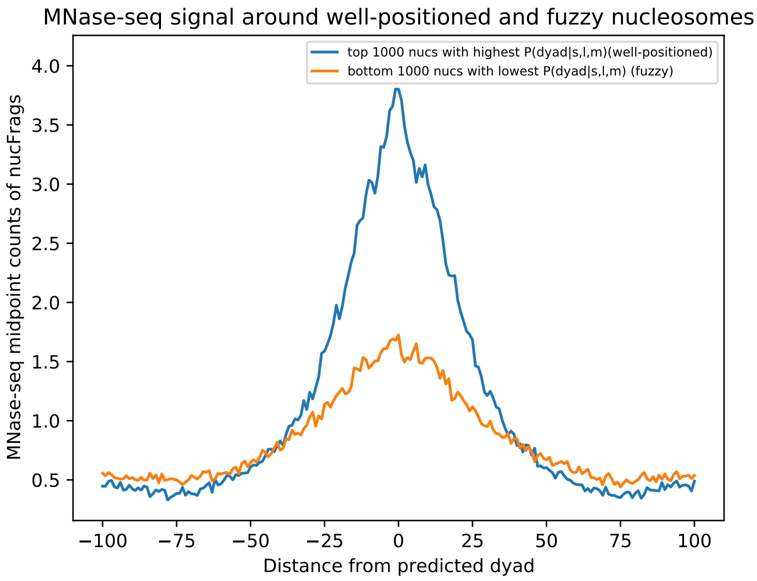

Supplementary Figure S3. The predicted dyad probability, P(dyad$|\boldsymbol{s},\boldsymbol{l},\boldsymbol{m}$), is a quantitative measure of the fuzziness of a nucleosome. The plot shows the MNase-seq midpoint counts of nucleosomal fragments separately for the top 1000 nucleosomes with the highest predicted dyad probability (blue) and the bottom 1000 nucleosomes with the lowest predicted dyad probability (orange). The midpoint counts are centered on the predicted nucleosome dyads. The signal around the top 1000 nucleosomes depict a sharper peak of midpoint counts of nucleosomal fragments indicating these to be well-positioned nucleosomes. In contrast, the signal for the bottom 1000 nucleosomes depict a shorter and slightly broader peak capturing the uncertainty in the positioning of the fuzzy nucleosomes.

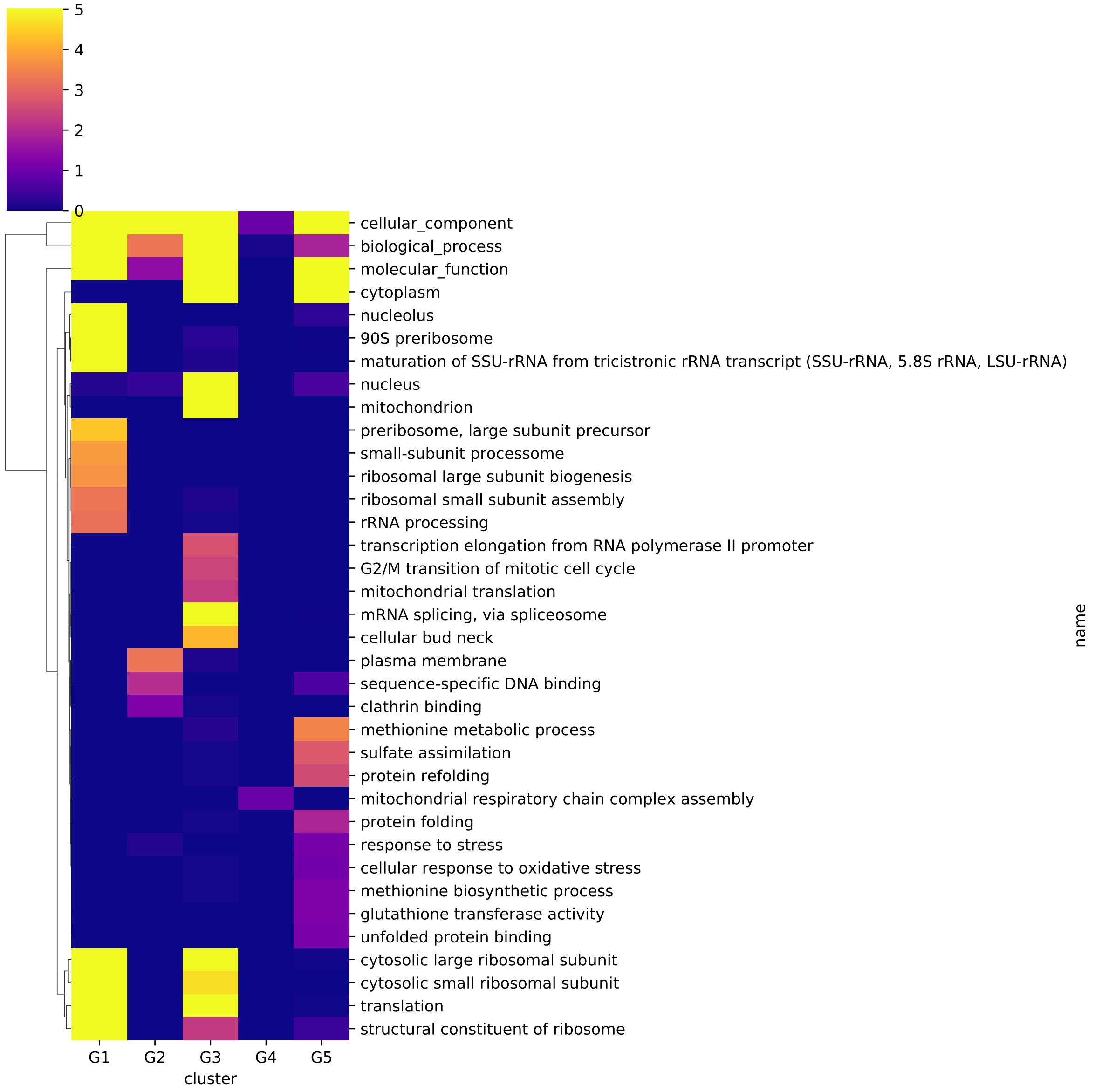

Supplementary Figure S4. Heatmap of GO terms obtained from genes in clusters found using $k = 5$ clusters. The top 15 hits per gene cluster with FDR $<$ 0.1 are selected for this plot. Colorbar depicts $-log_{10}FDR$ with a cutoff at 5.
